# Supervised learning for infection risk inference using pathology data

**DOI:** 10.1186/s12911-017-0550-1

**Published:** 2017-12-08

**Authors:** Bernard Hernandez, Pau Herrero, Timothy Miles Rawson, Luke S. P. Moore, Benjamin Evans, Christofer Toumazou, Alison H. Holmes, Pantelis Georgiou

**Affiliations:** 1 Centre for Bio-Inspired Technology, Department of Electrical and Electronic Engineering, Imperial College London, B422 Bessemer Building, South Kensington Campus, London, SW7 2AZ UK; 2 Health Protection Unit in Healthcare Associated infections and Antimicrobial Resistance, Imperial College London, 8th floor Commonwealth Building, Hammersmith Hospital Campus, Acton, London, W12 0NN UK

**Keywords:** Antimicrobial resistance, Infection, Machine learning, Supervised learning, Predictive modelling, Biochemical markers, Decision support, Behaviour change

## Abstract

**Background:**

Antimicrobial Resistance is threatening our ability to treat common infectious diseases and overuse of antimicrobials to treat human infections in hospitals is accelerating this process. Clinical Decision Support Systems (CDSSs) have been proven to enhance quality of care by promoting change in prescription practices through antimicrobial selection advice. However, bypassing an initial assessment to determine the existence of an underlying disease that justifies the need of antimicrobial therapy might lead to indiscriminate and often unnecessary prescriptions.

**Methods:**

From pathology laboratory tests, six biochemical markers were selected and combined with microbiology outcomes from susceptibility tests to create a unique dataset with over one and a half million daily profiles to perform infection risk inference. Outliers were discarded using the inter-quartile range rule and several sampling techniques were studied to tackle the class imbalance problem. The first phase selects the most effective and robust model during training using ten-fold stratified cross-validation. The second phase evaluates the final model after isotonic calibration in scenarios with missing inputs and imbalanced class distributions.

**Results:**

More than 50% of infected profiles have daily requested laboratory tests for the six biochemical markers with very promising infection inference results: area under the receiver operating characteristic curve (0.80-0.83), sensitivity (0.64-0.75) and specificity (0.92-0.97). Standardization consistently outperforms normalization and sensitivity is enhanced by using the SMOTE sampling technique. Furthermore, models operated without noticeable loss in performance if at least four biomarkers were available.

**Conclusion:**

The selected biomarkers comprise enough information to perform infection risk inference with a high degree of confidence even in the presence of incomplete and imbalanced data. Since they are commonly available in hospitals, Clinical Decision Support Systems could benefit from these findings to assist clinicians in deciding whether or not to initiate antimicrobial therapy to improve prescription practices.

## Background

Antimicrobials are drugs that kill or stop the growth of microbes (e.g. bacteria or viruses), thereby are commonly used to treat infections. Since their discovery in 1930s, the antimicrobial research community was concerned about their misuse and the possible consequences that could arise. Despite all their efforts to disseminate general awareness, Antimicrobial Resistance (AMR) has been reported to be a leading public health and safety threat [1, 2] with the inappropriate use of antimicrobials in humans as one of the leading drivers [3]. New diagnostic devices are being designed to detect infections, but they are still highly specific, expensive and slow; obstructing their adoption in hospital settings [4]. In scenarios where clinicians suspect infection, concerns over the management of the individual often promote a conservative therapy (e.g. broad spectrum antibiotics) before the results of diagnostics tests are available. Such behaviour, focusing only on the patient and not considering the long term consequences of prescribed therapies, promotes the misuse of antimicrobials and contributes to AMR [5–7].

Clinical Decision Support Systems (CDSSs) are used widely to improve quality of care by promoting behavioural change among clinicians in specific aspects such as prescribing [8]. They can be defined as a computer program designed to analyse data to help health care professionals make clinical decisions. Most basic systems include assessment, monitoring and informative tools in the form of computerized alerts, reminders and electronic clinical guidelines [9]. More advanced diagnosis and advisory tools usually rely on statistics and machine learning to provide a higher level of data abstraction for therapy advice [10] or risk assessment [11].

Over the last decade, there has been a significant surge of interest in using clinical data for decision support and therefore data mining and machine learning have been widely applied for knowledge discovery in medicine [12, 13]. This information is available in a variety of formats including lab results, clinical observations, imaging scans, free text notes and more. In particular, pathology laboratory tests for a few biochemical markers are commonly requested by practitioners on patient admission to hospital and at regular intervals during the stay of the patient. Therefore, it represents a rich resource of observational data with the potential to facilitate assessment and detection of infectious diseases, even at early stages.

A binary classifier is a computational model that predictively divides a dataset into two groups, positives and negatives. They have been successfully applied to medical problems in recent years. For instance, Decision Tree Classifiers (DTC) are popular for their simplicity to understand and construct from logical rules [14]. Single and ensemble decision trees have been applied to pathology laboratory data to enhance the diagnosis of infections caused by *Chlamydia pneumoniae* [15] and Hepatitis B/C viruses [16]. However, these studies used a relatively high number of variables per patient (16 and 18) and discarded those in which inputs were missing, reducing the size of the datasets considerably (1495 and 10378 observations respectively). Consequently, the accuracy of these tools was reported to be as low as 60–65%.

Another approach relies on Bayesian Networks which represent a set of variables (nodes) and their dependencies (arcs) using a graph. They have been used to predict bacteremia using 214 clinical variables [10]. The designed graph was utterly complex and provided an area under the receiver operating characteristic curve (AUCROC) of only 0.68. Furthermore, a comparison of machine learning methods for neonatal sepsis detection [17] in 299 infants using on average 17 clinical variables presented an AUCROC within the range 0.57–0.65. The imbalance between sensitivity and specificity metrics was also acutely problematic.

The integration of previous approaches in CDSSs is restrained for three main reasons: (i) the studies are focused on a single microbe; (ii) only blood infection (i.e. sepsis) was targeted and (iii) the collection of such high number of variables is laborious, if not intractable. This paper retrospectively evaluates the performance of different binary classifiers to detect any type of infection from a reduced set of commonly requested clinical measurements.

## Method

### Selected pathology biochemical markers

After reviewing the scientific literature and discussion with infectious disease experts, six routinely requested biomarkers were selected (see Table 1) which are deemed to provide sufficient information to evaluate the infection status of a patient by an expert physician. Note that not all biomarkers are directly related to infection (e.g. creatinine), however, previous studies have demonstrated a relationship between these biomarkers and infections [18, 19].

**Table 1:** Selected laboratory biochemical markers

Abbreviation	Marker	Unit
ALT	Alanine aminotransferase	iu/L
ALP	Alkaline phosphatase	iu/L
BIL	Bilirubin	umol/L
CRE	Creatinine	umol/L
CRP	C-Reactive protein	mg/L
WBC	White blood count	10*9/L

### Selected supervised learning models

Supervised learning is the area of machine learning that involves defining a mapping between data and an output label [20]. Well-known supervised machine learning algorithms for binary classification, restricted to those able to provide a probability outcome, were evaluated and compared. Not all classifiers provide probabilities inherently (e.g. Support Vector Machines) but additional algorithms exist to estimate them. A brief summary of the selected algorithms is presented below.

#### Gaussian Naïve Bayes

GNB is based on applying Bayes’ theorem with the assumption of independence between every pair of features. The likelihood function for each feature is assumed to be Gaussian and despite this simplifying assumption, it has worked quite well in many real-world situations (e.g. spam filtering) [21]. In addition, they require a small amount of training data to estimate the necessary parameters, are extremely fast compared to more sophisticated methods and the generated models can perform online updates.

#### Decision tree classifier

DTC is a simple algorithm for classifying observations based on recursive partitioning given an attribute value. They have been used in clinical domains since they are easy to interpret and understand. Furthermore, the time required to train them on large datasets is still reasonable. However, they do not tend to work well if decision boundaries are smooth; that is, significant overlap between categories. Also, as a result of the greedy strategy applied, they present high variance and are often unstable, tending to over-fit.

#### Random forest classifier

RFC is an ensemble learning method for classification based on DTCs. It constructs a set of DTCs trained with different portions of the data and outputs the class that is the mode of all the classifiers. They often correct the DTCs habit of over-fitting the training set.

#### Support vector machine

SVM uses a kernel function to transform the training samples to a new space with higher dimensionality [22]. The boundary found in the high dimensional space is the hyperplane which maximizes the distance between classes (i.e. maximum margin hyperplane) and can have a non linear shape in the original data space. It employs the principle of Structural Risk Minimization to generalize better than conventional machine learning methods which employ Empirical Risk Minimization [23]. Though SVMs do not directly provide probability estimates, they may be calculated in the binary case using Platt scaling; that is, logistic regression on the SVM’s scores [24].

### Assembling data for infection inference

**Fig. 1:**
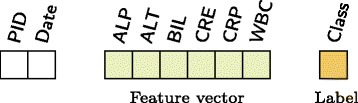
Profile with metadata, feature vector and label

In hospitals, data is compartmentalized with many distinct measurements of patient health being stored separately. In this paper, pathology and microbiology data for patients from all hospital wards at Imperial College Healthcare NHS Trust were extracted. In the absence of a single database linking pathology with microbiology data, these two different data sources were combined to create a unique dataset of profiles to perform detection of infection. Each profile (see Fig. 1) has the daily symptoms of a patient represented by six selected laboratory tests (constituting a patient’s feature vector), the infection condition extracted from the microbiology data (Label) and additional information for further data cleaning such as the patient identification number (PID).

Unfortunately, labels collected in such databases are recorded for purposes other than retrospective data analysis and it is difficult to define a “ground truth”. Initially, all profiles were labelled as culture-negative (C-). Then, any profile available for a patient with less than two days difference from a positive culture was assigned to the culture-positive (C+) category. This assumption comes from antimicrobial susceptibility tests taking from 24 to 48 hours and antibiotics often needing a period of time to kill or stop bacterial growth. Assigning profiles to the culture-negative category by default clearly produces mislabelled data. To tackle this issue, profiles within those periods of time in which there is no culture evidence (results for microbiology cultures are missing) are discarded. In addition, culture-negative profiles were removed if culture-positive profiles where present in a single patient admission.

### Challenges in clinical data: preprocessing

In machine learning applications, data preprocessing is a common step that becomes critical when dealing with data obtained from clinical environments. First, class imbalance must be tackled since unequal class distributions arise naturally. Also, data corruption is frequent [25] which can be classified as erroneous data, missing data and imprecise data. The steps followed in data preprocessing are briefly explained below.

#### Detection of outliers

The importance of outlier removal to develop robust predictive models has been demonstrated previously [26]. In our data, outliers are mainly caused by two main factors: susceptibility tests not requested or wrongly reported (human errors) and inaccurate microbiology results (diagnostic device errors or limitations). To identify and discard them the inter-quartile range rule (IQRxT) is applied to each category independently where T represents the threshold parameter. A threshold of T=1.5 is widely accepted and T=3 is considered to discard only extreme outliers.

#### Dealing with missing data

A large proportion of profiles are incomplete; that is, they do not have results for the six selected biomarkers. The notation *F*_*n*_ is used to define the fraction of data in which profiles have exactly *n* biomarkers. Exclusively complete profiles are manipulated to generate the predictive models while incomplete profiles $\left (\{F_{n}\}_{n=1}^{5}\right)$ are used to evaluate the robustness of such models for different degrees of missing variables. The statistical measure preferred for inputation of missing values is the median.

#### Dealing with class imbalance

The issue of class imbalance has been addressed by under-sampling the majority class (RAND_U_), over-sampling the minority class (RAND_O_) and using Synthetic Minority Over-sampling (SMOTE) [27] which blends both sampling methods to build classifiers with better performance.

#### Data scaling

Since data scaling is a common requirement for many machine learning algorithms and can favourably affect model performance, two approaches have been considered: (i) data normalization which scales individual features to have unit form and (ii) data standardization which transforms features so they are normally distributed (zero mean and unit variance).

### Evaluating performance for model selection

Initially, the data is divided into cross-validation (CVS) and hold-out sets (HOS) where the latter contains 25% of all observations. The CVS is manipulated to train and calibrate the models where data sampling and preprocessing should always be performed within cross-validation and using exclusively the observations within the training set. Applying them (particularly sampling) before cross-validation is a common malpractice for two main reasons: it leads to over-fitting problems, but more importantly it generates artificial observations (non real data) which are used in the testing fold for validation. As an example, RAND_O_ just duplicates entries and therefore the same observations would be seen during training and testing, defeating the whole purpose of cross-validation.

**Fig. 2:**
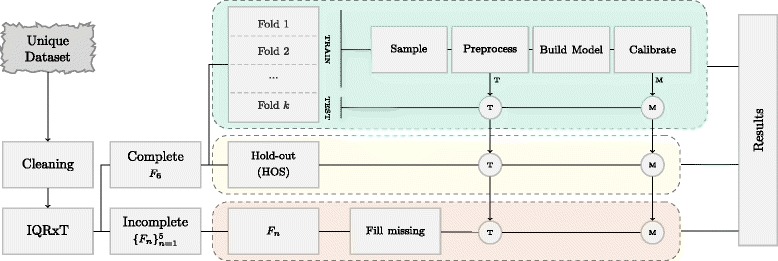
High level diagram of the work-flow followed to build the models and obtain the results presented in this paper. First, data cleaning and outlier removal is performed. The remaining observations are grouped as complete or incomplete profiles. The former is further split into Cross-Validation Set (CVS) and Hold-out Set (HOS). Ten-Fold Stratified Cross-Validation is performed on CVS and two outputs are obtained in this step: a preprocessing equation to transform new observations (T) and a calibrated model (M) which are later used. It is important to highlight that sampling and preprocessing are performed using the training set while calibration is achieved from completely unseen observations. The performance of calibrated models is evaluated in HOS and $\{F_{n}\}_{n=1}^{5}$

Ten-Fold Stratified Cross-Validation has been used in this paper to assess how well the classifiers will generalize to an independent data set (see Fig. 2). Firstly, the training set is sampled, preprocessed and used to build the model. As outputs, we obtain a preprocessing equation that will be applied to new observations and a non-calibrated model. Models are validated using both imbalanced and balanced (applying RAND_U_) versions of the testing fold to ensure it is performing appropriately (not over-fitting). Finally, to assess the translational utility of this results into a clinical decision support system, models are calibrated and validated in HOS and $\{F_{n}\}_{n=1}^{5}$ with observations that are completely unseen during data sampling/preprocessing and model training/calibration.

### Model calibration

Properly calibrated classifiers provide a probability which can be directly interpreted as a confidence interval. In binary classification, among the samples to which a calibrated model gave a probability close to 0.8, approximately 80% actually belong to the positive class. Some models (e.g. Logistic Regression) return well calibrated predictions by default while others introduce bias (e.g. GNB pushes probabilities to 0 or 1). They can be calibrated using a dataset not seen during training [28]. In this paper isotonic calibration was selected.

### Evaluation metrics

There are many different metrics for assessing the performance of classifiers [29, 30]. For binary classifiers, most of them are based on four simple measures: the number of true positives (TP), the number of false positives (FP), the number of true negatives (TN) and the number of false negatives (FN). Sensitivity, specificity and overall accuracy are commonly used to demonstrate classifiers performance. Note however, that accuracy might not be appropriate when the class sizes differ considerably [31]. For detailed information of classifiers, receiver operating characteristic (ROC) and precision-recall (PR) curves are often presented [32, 33]. The ROC curve is created by representing the true positive rate against the false positive rate for different threshold settings while the PR curve represents precision against recall. The area under such curves is commonly used for comparison. It is important to mention that precision is affected by class proportions, and hence PR is conditioned too. On the contrary, sensitivity, specificity and ROC are agnostic to class proportions. The definition and equations of previously mentioned metrics are shown in Table 2.

**Table 2:** Evaluation metrics: descriptions and equations

Metric	Description	Equation
Sensitivity	Proportion of observed positives that are correctly identified as such (i.e. percentage of culture-positive profiles correctly identified as positive). Also called recall (REC) or true positive rate (TPR).	$SENS=\frac {TP}{TP+FN}$
Specificity	Proportion of observed negatives that are correctly identified as such (i.e. percentage of culture-negative profiles correctly identified as negative). Also called true negative rate (TNR).	$SPEC=\frac {TN}{TN+FP}$
ROC	This curve illustrates the performance of a binary classifier as its discrimnation threshold is varied by plotting true positive rate (TPR) against false positive rate (FPR). It is related to cost/benefit analysis of diagnostic decision making.	
PR	This curve represents precision against recall where high scores for both shows that the classifier is returning accurate results (high precision) as well as returning a majority of all positive results (high recall).	

### Statistical analysis

The statistical significance of the differences between the classifiers was determined using the non-parametric test (Kruskal-Wallis one-way ANOVA on ranks) where the significance level was set at *p* < 0.05. Post-hoc analysis (Fisher’s LSD) was used to determine pairwise differences. Analyses were performed with NCSS version 8.

### Software

The Python programming language was used in this research. Supervised learning models and performance metrics from Scikit-learn [34] and sampling techniques from Imbalanced-learn [35] were employed. Data handling was done with Pandas [36, 37] and data visualization using Matplotlib [38] and Seaborn [39].

## Results

This study was conducted with data from the Imperial College Healthcare NHS Trust, which comprises three separate hospitals, totalling 1500 beds and serving a population of 2.5 million citizens. Combining pathology and microbiology records over two years (2014 and 2015) yielded over one and a half million profiles for more than half a million different patients. From these data, 43,497 (2.7%) profiles for 12,099 (2.1%) patients were assigned to the culture-positive category. Therefore, classes were clearly imbalanced with culture-negative constituting the majority.

### Data insights

#### Laboratory tests frequency

**Fig. 3:**
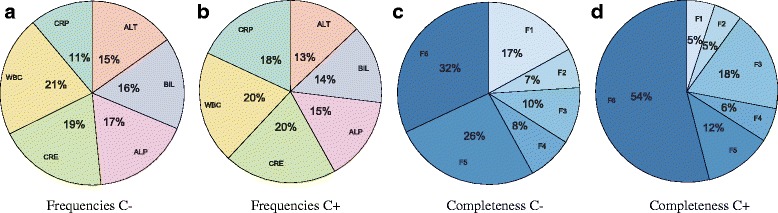
Percentages representing the frequency of each biomarker (**a** and **b**) and the completeness of profiles (**c** and **d**) for both culture-negative (C-) and culture-positive (C+) categories respectively

**Fig. 4:**
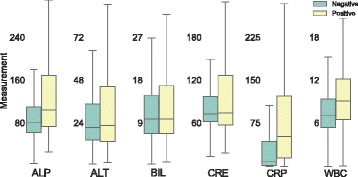
Distribution of measurements for each single biomarker grouped in two categories: culture-negative (C-) and culture-positive (C+). The inter-quartile range rule with threshold of 1.5 (IQRx1.5) has been applied to each category independently to discard outliers

The number of laboratory tests requested per biomarker is explained for both categories (culture-negative and culture-positive) independently in Table 3. The notation F is used to categorize profiles according to the number of biomarkers available. Hence, F_2_ contains all profiles with exactly two biomarkers. Obviously, some biomarkers are requested more frequently than others; from the instances presented in Table 3, the corresponding proportions are displayed for culture-negative (Fig. 3 a) and culture-positive (Fig. 3 b) categories. The most requested biomarkers are WBC and CRE for both categories. It is worth stressing that CRP is requested more frequently for infected patients, since it is often a good indicator of infection. Its presence is almost double; from 11% in culture-negative profiles to 18% in culture-positive profiles. Hence, although CRP would appear to be sufficient for infection detection by looking at its distribution (see Fig. 4), it presents two main issues: it is the least requested of all biomarkers in the culture-negative category (11%) and it does not provide any information regarding the location of the infection.

**Table 3:** Pathology biomarkers and profiles overview

		ALP	ALT	BIL	CRE	CRP	WBC	All Tests	Profiles
C-	F_1_	10858	236	327	53443	10477	191213	266554	266554
	F_2_	11654	492	889	81337	25959	94605	214936	107468
	F_3_	51047	27921	28506	131058	113049	130870	482451	160817
	F_4_	135450	97665	101738	112962	36446	59607	543868	135967
	F_5_	412266	386171	409873	404555	58530	391120	2062515	412503
	F_6_	517397	517397	517397	517397	517397	517397	3104382	517397
	Total	1138672	1029882	1058730	1300752	761858	1384812	**6674706**	**1600706**
C+	F_1_	40	5	7	412	267	1445	2176	2176
	F_2_	103	12	20	1458	1140	1983	4716	2358
	F_3_	484	85	121	7671	7367	7621	23349	7783
	F_4_	2395	373	578	2308	1946	2096	9696	2424
	F_5_	5277	3043	5145	5165	3106	4674	26410	5282
	F_6_	23474	23474	23474	23474	23474	23474	140844	23474
	Total	31773	26992	29345	40488	37300	41293	**207191**	**43497**

Bold numbers indicate total numbers of tests and profiles

#### Profile completeness

A common problem in previous studies was missing data leading to incomplete profiles. Therefore, the proportion of profiles with different levels of completeness is displayed in Fig. 3 c and d. More than 50% of the culture-positive profiles are complete; that is, contain results for the six biomarkers. In contrast, the percentage of complete profiles drops to 28% for the culture-negative category. Taking into consideration profiles with at least four biomarkers available $\left (\{F_{n}\}_{n=4}^{6}\right)$ increases percentages to 65% (C-) and 71% (C+); that is, approximately two thirds of all available profiles. Hence, it is important to identify classifiers that are able to infer infection likelihood for incomplete profiles to increase usability in real-life clinical decision support systems.

#### Distributions of selected biomarkers

The density distribution for each biomarker is presented in Fig. 4 for culture-positive and culture-negative categories. Most distributions are skewed (especially for C+) and robust measures for central tendency (median) and statistical dispersion (interquartile range) are used to describe them. Outliers were removed by applying the IQRx1.5 rule to both categories independently.

The distance between medians for each category is clearly noticeable for CRP and appreciable to a lesser extent in WBC and ALP. On the other hand, there is no perceptible difference between the medians of culture-positive and culture-negative profiles for the rest of the biomarkers (ALT, BIL, CRE). Regarding statistical dispersion, CRP presents a huge contrast between the two categories, followed by CRE and ALP. It is clear that most biomarkers have an overlapping region between both categories. Crearly, this is a very challenging area for infection risk inference that could be slightly ameliorated by relaxing the IQR threshold for the culture-positive category.

### Infection risk inference on complete profiles

A comparison of the best overall binary classifier for each supervised model is presented in Table 4 where metrics are evaluated on a balanced version of the hold-out set after isotonic calibration. Standardization performed consistently better than normalization and therefore only these results are presented. The first two columns indicate the sampling method and the algorithm evaluated respectively. The metric scores from left to right are: area under the ROC curve (AUCROC), area under the PR curve (AUCPR where the subscript B indicates that classes were balanced), sensitivity (SENS) and specificity (SPEC).

**Table 4:** Sampling method: performance comparison

		AUCROC	AUCPR_B_	SENS	SPEC
RAND_U_	GNB	0.763	0.871	0.533	0.992
	DTC	0.798	0.891	0.601	0.993
	RFC	0.791	0.892	0.583	0.993
	SVM	0.792	0.894	0.593	0.991
RAND_O_	GNB	0.742	0.860	0.482	0.991
	DTC	0.810	0.876	0.688	0.932
	RFC	0.801	0.901	0.617	0.990
	SVM	0.753	0.872	0.523	0.991
SMOTE	GNB	0.814	0.872	0.725	0.903
	DTC	0.779	0.881	0.636	0.963
	RFC	0.818	0.876	0.725	0.909
	SVM	0.830	0.884	0.747	0.912

The performance of the classifiers can be seen to vary according to the sampling technique used. In particular, the classifiers generated using SMOTE present the highest sensitivities. It is particularly notable for the GNB classifier in which it rises from 0.482 (random over-sampling) and 0.533 (random under-sampling) to a value of 0.725 when SMOTE is applied. This boost in sensitivity leads to an increase in the AUCROC to 0.814. The SVM classifier achieves a slightly better performance with an AUCROC of 0.830. Furthermore, both classifiers present equilibrium between sensitivity and specificity which can be quantified by the Geometric-Mean; 0.809 (GNB) and 0.825 (SVM). The performance of tree-based methods does not change significantly among sampling techniques, probably due to over-fitting. Note that DTC presents the highest sensitivity when culture-positive observations are merely duplicated using RAND_O_.

In further analysis, only models generated using the SMOTE sampling technique and isotonic calibration are considered. The models selected are: (i) GNB with priors of 0.5, since categories are balanced (ii) DTC with a minimum number of samples in a leaf of 50 and a minimum number of observations in a node in order to be split of 200 (iii) RFC with 10 estimators (trees) (iv) SVM with penalty factor of *C*=1.0 and radial basis kernel where *γ*=0.1.

### Infection risk inference on incomplete profiles

The behaviour of the selected models for different degrees of missing inputs is compared in Table 5. Since they were trained on complete profiles (F_6_) and the biomarkers distributions are non-symmetrical (see Fig. 4) the statistical measure preferred to input missing values is the median. In particular, the median for each biomarker is extracted from the observations used to train the model where both categories (C+ and C-) are balanced.

**Table 5:** Missing data: performance comparison

		AUCROC	AUCPR_B_	SENS	SPEC
GNB	F_6_	0.814	0.872	0.725	0.903
	F_5_	0.802	0.874	0.664	0.939
	F_4_	0.803	0.874	0.669	0.938
	F_3_	0.750	0.832	0.589	0.912
	F_2_	0.686	0.816	0.400	0.971
	F_1_	0.569	0.767	0.145	0.994
DTC	F_6_	0.799	0.881	0.636	0.963
	F_5_	0.777	0.859	0.614	0.940
	F_4_	0.769	0.839	0.652	0.886
	F_3_	0.702	0.777	0.672	0.732
	F_2_	0.617	0.722	0.583	0.652
	F_1_	0.535	0.684	0.480	0.590
RFC	F_6_	0.818	0.876	0.725	0.909
	F_5_	0.806	0.874	0.682	0.930
	F_4_	0.805	0.867	0.707	0.903
	F_3_	0.764	0.826	0.707	0.822
	F_2_	0.704	0.796	0.504	0.904
	F_1_	0.599	0.775	0.212	0.987
SVM	F_6_	0.830	0.884	0.747	0.912
	F_5_	0.816	0.885	0.687	0.944
	F_4_	0.809	0.874	0.694	0.924
	F_3_	0.768	0.837	0.654	0.881
	F_2_	0.699	0.809	0.453	0.949
	F_1_	0.591	0.785	0.186	0.996

**Fig. 5:**
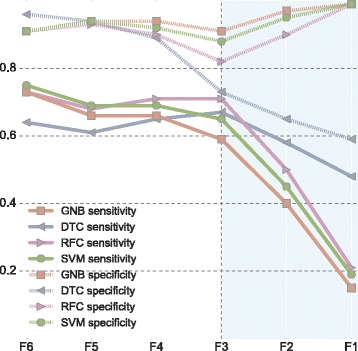
Sensitivity and specificity variation for different degrees of missing inputs

The scores obtained for $\{F_{n}\}_{n=4}^{6}$ are very similar and indicate that all classifiers perform without noticeable loss in performance if at least four biomarkers are available. This is observable in Fig. 5 where sensitivity (solid line) and specificity (dashed line) from Table 5 have been graphically represented. Furthermore, the results obtained for F_3_ are slightly inferior and the main drop in performance materializes for F_2_ indicating insufficient information to perform infection inference. This is noticeable primarily in the sensitivity score. In addition, there is a clear trade-off between sensitivity and specificity where the former represents a main barrier for incomplete profiles. As mentioned previously, the behaviour of DTC is the least reliable. In this case, it shows an unexpected increase in sensitivity when data is missing, likely due to algorithm propensity to overfit, with a maximum of 0.672 for F_3_. The use of an ensemble approach (RFC) corrected this issue. The best balance between sensitivity and specificity is obtained by the SVM where the former is the highest amongst all algorithms (0.747) and is robust to incomplete inputs. Also, it presents the highest AUCROC (0.830). Since AUCROC and SENS are statistically significant across classifiers (*p*-values < 0.01), the SVM has been selected for further analysis.

### Understanding the behaviour of the predictive model

**Fig. 6:**
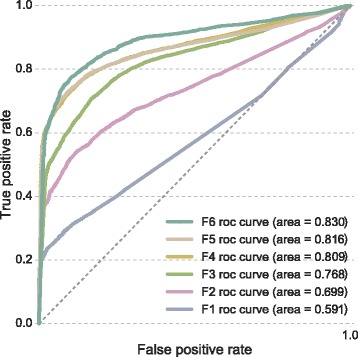
ROC curves for the selected SVM classifier on different degrees of missing inputs. F4 indicates that four inputs are available

**Fig. 7:**
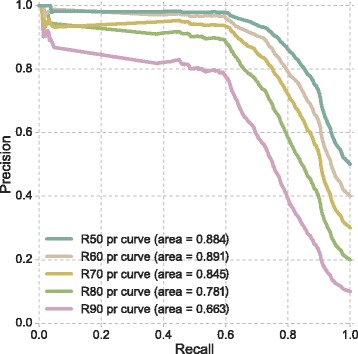
PR curves for the selected SVM classifier on different degrees of imbalanced categories. R60 indicates that 60% of the observations are culture-negative

In order to understand the response of the selected model (SVM classifier) in real clinical settings, two different types of scenarios have been considered: missing inputs and imbalanced class distributions. The former has been assessed through the ROC curves presented in Fig. 6. As expected from previous results, curves obtained for $\{F_{n}\}_{n=4}^{6}$ are quite similar with an AUCROC of approximately 0.8. Furthermore, they exhibit an appropriate trade-off between specificity and sensitivity. Since the classifier is intended to operate in scenarios where class imbalance is common, the PR curves are shown in Fig. 7. Note that the ROC curve is a good indicator of overall performance but does not reflect the effect of class imbalance. The notation R80 indicates that 80% of observations belong to the culture-negative category. In scenarios with balanced classes the predictive model shows a good balance between precision and recall and an AUCPR of 0.884. The model is robust against class imbalance and the drop in AUCPR occurs for scenarios with the imbalance ratio of 1/9 (90%) or higher.

**Fig. 8:**
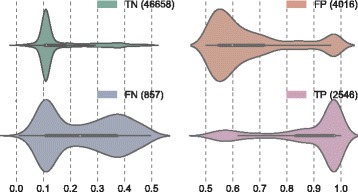
Probability density distributions for each type of prediction in the confusion matrix: TP, TN, FP and FN

For further understanding of the probabilities provided by the predictive model in an extremely imbalanced scenario, a total of 54077 observations were tested (94% belonging to C- and 6% to C+ approximately). The instances and density distribution for each type of classification (true positive, true negative, false positive and false negative) are shown in Fig. 8. A discrimination threshold of 0.5, commonly used in binary classifiers, has been applied to assign the predicted category (C- or C+).

Firstly, it is important to notice that extreme probabilities generally correspond to correct predictions. In particular, the probability ranges for true negative and true positives are [0.1,0.2] and [0.85,1.0] respectively. The number of false positives looks extremely high but it is due to such acute class imbalance. Only 255 false positives were obtained in a balanced scenario. Furthermore, this type of error is easily identifiable since their probabilities lie mostly within the range [0.55,0.7] without considerable overlapping. The density distribution for the false negative predictions is spread across the range [0.1,0.4] and overlaps slightly with the true negatives distribution. The probabilities are distributed evenly and might correspond to sporadic situations such as very early stage infections in which symptoms are still not clear or cases in which the correct therapy has been applied and therefore pathology biomarkers have been properly controlled.

## Discussion

In infectious diseases, antibiotic selection has been the main focus of Clinical Decision Support Systems (CDSSs) [40]. However, improving antibiotic selection does not necessarily imply a reduction in antibiotic prescription, it might even encourage it. Therefore, assisting clinicians by providing the risk of infection for an individual patient, and on whether or not to initiate antibiotic therapy, can potentially reduce the misuse of antibiotics. The main reasons obstructing inclusion in CDSSs were: (i) studies were highly specific by tackling individual microbes and single infections (sepsis is the most common) (ii) they required a high number of variables whose collection is laborious (iii) scenarios with missing data, which are very common in clinical environments, were completely ignored (iv) there was a lack of thorough description and evaluation of the models to understand their behaviour and support confidence.

### Selection of clinical features

The first challenge while designing a model for classification is deciding which input parameters are to be considered. Based on the recommendations of infection specialists and clinicians, six generic biochemical markers were selected which were found to be available, especially for infected patients, on a daily basis. To diagnose bacterial infections, Procalcitonin (PCT) has presented slightly better diagnostic accuracy than C-Reactive Protein (CRP) [41]. However, CRP was requested considerably more often in our data and therefore was favoured for inclusion in the study.

Liver failure is known to be associated with increased risk of infection and therefore culture-positive samples [42]. Among the biochemical markers commonly examined by clinicians to diagnose liver failure, we have considered Alanine aminotransferase (ALT), Alkaline phosphatase (ALP) and biliribin (BIL). In addition, culture-positive samples are associated with high severity scores (SAPS II > 43 and SOFA > 4) [42]. From the large amount of clinical features required to compute such scores, bilirubin (BIL), white blood cell counts (WBC) and creatinine (CRE) are common to this study. Since some of the selected biochemical markers have different normal reference ranges according to age and gender, the inclusion of such variables could potentially increase the accuracy of the classifiers.

### Addressing class imbalance

As commonly expected from clinical data, categories were clearly imbalanced and different sampling techniques were explored to tackle this issue. Simple methods such as random under-sampling and/or over-sampling have proven to be valid in other domains. Undoubtedly, choosing an adequate sampling technique depends on the data, but it is clear that under-sampling potentially discards useful information and over-sampling replicates observations which might lead to over-fitting. In fact, Synthetic Minority Oversampling Technique (SMOTE) proved to be a better approach which outperformed previous techniques and enhanced the sensitivity of the generated models.

### Effect of missing inputs in prediction

Unfortunately, missing variables is a common problem in clinical data. Since this is a retrospective study, we have to deal with the fact that the data were not collected to generate a predictive model. For these reasons, it is highly desirable for a classification system to be robust to incomplete inputs. The SVM classifier is robust and operates without noticeable loss in performance if at least four biomarkers are present. DTCs are widely used in clinical research and the results obtained in this paper outperform those presented in similar studies [15, 16]. However, this method is the most affected by missing biomarkers as a result of the greedy strategy applied. In previous studies RFC was selected as an ensemble method based on DTCs [15–17] to tackle this issue. The unexpected increase in sensitivity presented by DTC for scenarios with missing data was corrected. However, performance was found to be similar.

### Selecting a suitable algorithm

From the obtained results, infection inference is feasible using only the six selected biomarkers with an AUCROC of approximately 0.8. In addition, sensitivity and specificity were both high and balanced in comparison to previous studies [17]. The best performance corresponds to a SVM classifier with penalty factor of *C*=1.0 and radial basis kernel where *γ*=0.1. The main disadvantage of this method is the large amount of computational resources (memory and time) required. Conversely, despite the simplicity of Gaussian Naïve Bayes (GNB), the difference in performance compared to complex algorithms is minimal. It also has additional desirable properties, namely that it requires a small amount of training data, it is very computationally efficient and performs online updates. These results were obtained from real observations (not synthetically generated) which were completely unseen during sampling, preprocessing and model calibration. The latter is often ignored but necessary to guarantee that probabilities use the whole spectrum [0,1] and are informative by providing the degree of confidence in the prediction. The Bootstrap aggregating technique was explored to build ensemble classifiers based on GNB and SVM, but it did not provide any significant improvement.

### Translational utility

It is important to recognize that the evaluation in the training phase is different from the evaluation of the final model. The first phase is to tune the models’ hyperparameters and select the most effective and robust model during training. The second phase is to evaluate the final model after the training. Ideally, the test data of this phase reflects the class distributions of the original population even though such distributions are usually unknown. Since the SVM classifier presented a robust response (and the highest sensitivities) in scenarios with missing data and imbalanced categories, it has been selected for further inclusion in the EPIC IMPOC (Enhanced Personalized and Integrated Care for Infection Management at Point of Care) decision support system to assist clinicians [43].

### Limitations

Profiles were assigned to the culture-positive (C+) category based on evidence of organism growth in the microbiology samples. Since there was a lack of no-growth evidence, remaining profiles were assigned to the culture-negative (C-) category. This limitation was tackled through data cleaning and outlier detection. However, providing no-growth evidence could boost performance even further. Also note that all patients and possible types of infection encountered in the hospital were considered.

## Conclusion

In this study, we have shown that it is feasible to perform infection inference using six biomarkers with a high degree of confidence (AUCROC > 0.8). To improve antibiotic prescribing and reduce patients’ unnecessary exposure to antibiotics in hospitals, new mechanisms for supporting clinicians decision making are urgently required. Using our selected biomarkers, enough information was available on a daily basis to perform such inference, even in the presence of incomplete and imbalanced data. The SVM model (*C*=1.0 and radial basis kernel with *γ*=0.1) was isotonically calibrated and thoroughly evaluated by mimicking a wide range of conditions (some of them extreme) in which the classifier would operate. Its response was robust and validated for translational utility. An empirical study to quantify the costs of different mistakes (false positives and false negatives) to understand their consequences and effects on clinicians prescription practices forms the basis of our future work. In addition, missing data will be handled more efficiently by finding correlations between biomarkers to determine more suitable values other than the median. With further integration in a decision support system, this work holds promise of alleviating inadequate prescription practices to enhance infection management and contribute to halting the progression of AMR.

## Abbreviations

ALP: Alkaline phosphatase
ALT: Alanine aminotransferase
AMR: Antimicrobial resistance
AUCPR: Area under curve precision-recall
AUCROC: Area under curve receiver operating characteristic
BIL: Bilirubin
C+/C-: Culture positive/negative
CDSS: Clinical decision support system
CRE: Creatinine
CVS: Cross-validation set
DTC: Decision tree classifier
FN: False negatives
FP: False positives
GNB: Gaussian Naï
ve Bayes; HOS: Hold-out set
IQR: Inter-quartile range
PID: Personal identification number
PR: Precision-recall
RAND_O_: Random oversampling
RAND_U_: Random undersampling
RFC: Random forest classifier
ROC: Receiver operating characteristic
SMOTE: Synthetic minority oversampling technique
SENS: Sensitivity
SPEC: Specificity
SVM: Support vector machine
TN: True negatives
TP: True positives
WBC: White blood count

## References

[1] Wise R, Hart T, Cars O, Streulens M, Helmuth R, Huovinen P, Sprenger M (1998). Antimicrobial resistance is a major threat to public health. Br Med J.

[2] O’Neill J. Antimicrobial resistance: tackling a crisis for the health and wealth of nations. London: Review on Antimicrobial Resistance. 2014. p. 1–16.

[3] Holmes AH, Moore LS, Sundsfjord A, Steinbakk M, Regmi S, Karkey A, Guerin PJ, Piddock LJ (2016). Understanding the mechanisms and drivers of antimicrobial resistance. Lancet.

[4] Banoo S, Bell D, Bossuyt P, Herring A, Mabey D, Poole F, Smith PG, Sriram N, Wongsrichanalai C, Linke R (2008). Evaluation of diagnostic tests for infectious diseases: general principles. Nat Rev Microbiol.

[5] Byl B, Clevenbergh P, Jacobs F, Struelens MJ, Zech F, Kentos A, Thys JP (1999). Impact of infectious diseases specialists and microbiological data on the appropriateness of antimicrobial therapy for bacteremia. Clin Infect Dis.

[6] Harbarth S, Garbino J, Pugin J, Romand JA, Lew D, Pittet D (2003). Inappropriate initial antimicrobial therapy and its effect on survival in a clinical trial of immunomodulating therapy for severe sepsis. Am J Med.

[7] Willemsen I, Groenhuijzen A, Bogaers D, Stuurman A, van Keulen P, Kluytmans J (2007). Appropriateness of antimicrobial therapy measured by repeated prevalence surveys. Antimicrob Agents Chemother.

[8] Rawson TM, Moore LSP, Hernandez B, Charani E, Castro-Sanchez E, Herrero P, Hayhoe B, Hope W, Georgiou P, Holmes AH (2017). A systematic review of clinical decision support systems for antimicrobial management: Are we failing to investigate these interventions appropriately?. Clin Microbiol Infect..

[9] McGregor JC, Weekes E, Forrest GN, Standiford HC, Perencevich EN, Furuno JP, Harris AD (2006). Impact of a computerized clinical decision support system on reducing inappropriate antimicrobial use: a randomized controlled trial. J Am Med Inform Assoc.

[10] Paul M, Andreassen S, Nielsen AD, Tacconelli E, Almanasreh N, Fraser A, Yahav D, Ram R, Leibovici L, Group TS (2006). Prediction of bacteremia using treat, a computerized decision-support system. Clin Infect Dis.

[11] Mullett CJ, Thomas JG, Smith CL, Sarwari AR, Khakoo RA (2004). Computerized antimicrobial decision support: an offline evaluation of a database-driven empiric antimicrobial guidance program in hospitalized patients with a bloodstream infection. Int J Med Inform.

[12] Cleophas TJ, Zwinderman AH, Cleophas-Allers HI (2013). Machine Learning in Medicine.

[13] Lucas PJ, van der Gaag LC, Abu-Hanna A (2004). Bayesian networks in biomedicine and health-care. Artif Intell Med.

[14] Negnevitsky M (2005). Artificial Intelligence: a Guide to Intelligent Systems.

[15] Richardson AM, Hawkins S, Shadabi F, Sharma D, Fulcher J, Lidbury B, et al.Enhanced laboratory diagnosis of human Chlamydia pneumoniae through pattern recognition derived from pathology database analysis. In: Supplementary proceedings of the third IAPR International Conference on Pattern Recognition in Bioinformatics (PRIB 2008). Melbourne, Australia: 2008. p. 227–34.

[16] Richardson AM, Lidbury BA (2013). Infection status outcome, machine learning method and virus type interact to affect the optimised prediction of hepatitis virus immunoassay results from routine pathology laboratory assays in unbalanced data. BMC Bioinformatics.

[17] Mani S, Ozdas A, Aliferis C, Varol HA, Chen Q, Carnevale R, Chen Y, Romano-Keeler J, Nian H, Weitkamp JH (2014). Medical decision support using machine learning for early detection of late-onset neonatal sepsis. J Am Med Inform Assoc.

[18] Kim WR, Flamm SL, Di Bisceglie AM, Bodenheimer HC (2008). Serum activity of alanine aminotransferase (alt) as an indicator of health and disease. Hepatology.

[19] Sierra R, Rello J, Bailén MA, Benítez E, Gordillo A, León C, Pedraza S (2004). C-reactive protein used as an early indicator of infection in patients with systemic inflammatory response syndrome. Intensive Care Med.

[20] Mohri M, Rostamizadeh A, Talwalkar A (2012). Foundations of Machine Learning.

[21] Metsis V, Androutsopoulos I, Paliouras G (2006). Spam filtering with naive bayes-which naive bayes?. Third Conference on Email and Anti-Spam, CEAS 2006, Mountain View, California, USA.

[22] Hernandez B (2013). Multi-View Object Recognition and Classification. Graph-Based Representation of Visual Features and Structured Learning and Prediction.

[23] Shin H, Cho S. How to deal with large dataset, class imbalance and binary output in svm based response model. Proceedings of the Korean Data Mining Society Conference. 2003:93–107. Best Paper Award.

[24] Platt J (1999). Probabilistic outputs for support vector machines and comparisons to regularized likelihood methods. Adv Large Margin Classifiers.

[25] Johnson AE, Ghassemi MM, Nemati S, Niehaus KE, Clifton DA, Clifford GD (2016). Machine learning and decision support in critical care. Proc IEEE.

[26] Osborne JW, Overbay A (2004). The power of outliers (and why researchers should always check for them). Pract Assess Res Eval.

[27] Chawla NV, Bowyer KW, Hall LO, Kegelmeyer WP (2002). Smote: synthetic minority over-sampling technique. J Artif Intell Res.

[28] Niculescu-Mizil A, Caruana R (2005). Predicting good probabilities with supervised learning. Proceedings of the 22nd International Conference on Machine Learning (ICML) 2005, vol. 149.

[29] Bekkar M, Djemaa HK, Alitouche TA. Evaluation measures for models assessment over imbalanced datasets. J Inf Eng Appl.2013;3(10).

[30] Baldi P, Brunak S, Chauvin Y, Andersen CA, Nielsen H (2000). Assessing the accuracy of prediction algorithms for classification: an overview. Bioinformatics.

[31] He H, Garcia EA (2009). Learning from imbalanced data. IEEE Trans Knowl Data Eng.

[32] Fawcett T (2006). An introduction to roc analysis. Pattern Recogn Lett.

[33] Davis J, Goadrich M (2006). The relationship between precision-recall and roc curves. Proceedings of the 23rd international conference on Machine learning (ICML) 2006. vol, 148.

[34] Pedregosa F, Varoquaux G, Gramfort A, Michel V, Thirion B, Grisel O, Blondel M, Prettenhofer P, Weiss R, Dubourg V, Vanderplas J, Passos A, Cournapeau D, Brucher M, Perrot M, Duchesnay E (2011). Scikit-learn: Machine learning in Python. J Mach Learn Res.

[35] Lemaître G, Nogueira F, Aridas CK (2017). Imbalanced-learn: A python toolbox to tackle the curse of imbalanced datasets in machine learning. J Machine Learning Research.

[36] McKinney W. pandas: a foundational python library for data analysis and statistics. Python for High Performance and Scientific Computing. 2011:1–9.

[37] McKinney W, et al. Data structures for statistical computing in python. In: Proceedings of the 9th Python in Science Conference. vol. 445: 2010. p. 51–6.

[38] Hunter JD (2007). Matplotlib: A 2d graphics environment. Comput Sci Eng.

[39] Waskom M, Botvinnik O, drewokane, Hobson P, David, Halchenko Y, Lukauskas S, Cole JB, Warmenhoven J, de Ruiter J, Hoyer S, Vanderplas J, Villalba S, Kunter G, Quintero E, Martin M, Miles A, Meyer K, Augspurger T, Yarkoni T, Bachant P, Williams M, Evans C, Fitzgerald C, Brian, Wehner D, Hitz G, Ziegler E, Qalieh A, Lee A. seaborn: v0.7.1 (June 2016). 2016. doi: doi:10.5281/zenodo.54844.

[40] Leibovici L, Paul M, Nielsen AD, Tacconelli E, Andreassen S (2007). The treat project: decision support and prediction using causal probabilistic networks. Int J Antimicrob Agents.

[41] Nargis W, Md I, Ahamed BU (2014). Procalcitonin versus C-reactive protein: Usefulness as biomarker of sepsis in ICU patient. International Journal of Critical Illness and Injury Science.

[42] Previsdomini M, Gini M, Cerutti B, Perren A, Dolina M (2012). Predictors of positive blood cultures in critically ill patients: a retrospective evaluation. Croatian Medical Journal.

[43] Hernandez B, Herrero P, Rawson TM, Moore LSP, Charani E, Holmes AH, Georgiou P (2017). Data-driven Web-based Intelligent Decision Support System for Infection Management at Point-Of-Care: Case-Based Reasoning Benefits and Limitations. Proceedings of the 10th International Joint Conference on Biomedical Engineering Systems and Technologies - Volume 5: HEALTHINF, (BIOSTEC 2017).

